# Effects of group housing and incremental hay supplementation in calf starters at different ages on growth performance, behavior, and health

**DOI:** 10.1038/s41598-022-07210-7

**Published:** 2022-02-24

**Authors:** Fatemeh Ahmadi, Ebrahim Ghasemi, Masoud Alikhani, Majid Akbarian-Tefaghi, Morteza Hosseini Ghaffari

**Affiliations:** 1 Department of Animal Sciences, College of Agriculture, Isfahan University of Technology, Isfahan, 84156–83111 Iran; 2 Physiology and Hygiene Unit, Institute of Animal Science, University of Bonn, 53115 Bonn, Germany

**Keywords:** Ageing, Nutrition

## Abstract

The present study examined the effects of age at group housing and age at incremental hay supplementation in calf starters from 7.5 to 15% (dry matter, DM) and their interaction on growth performance, behavior, health of dairy calves, and development of heifers through first breeding. A total of 64 calves (n = 16 calves/treatment, 8 male and 8 female) were randomly assigned to 4 treatments in a 2 × 2 factorial arrangement, with age at group housing (early = d 28 ± 2, EG vs. late = d 70 ± 2, LG; 4 calves per group) and age at incremental hay supplementation of calf starters from 7.5 to 15% of DM (early = d 42 ± 2 d, EH vs. late = d 77 ± 2, LH) as the main factors. All calves (female and male) were weaned at 63 days of age and observed until 90 days of age. Heifer calves were managed uniformly from 90 days of age until first calving to evaluate the long-term effects of treatment. No interactions were observed between age at group housing and age at incremental hay to calves on starter feed intake, performance, calf health and behavior, and heifer development through first breeding, which was contrary to our hypothesis. The age at which incremental hay supplementation was administered had no effect on starter feed intake, growth performance, or heifer development until first calving. When EG calves were compared with LG calves, nutrient intake (starter, total dry matter, metabolizable energy, neutral detergent fiber, starch, and crude protein), average daily gain, and final body weight increased. In addition, frequency of standing decreased and time and frequency of eating increased in EG calves compared to LG calves. Overall, early group housing leads to improved growth performance in dairy calves with no negative effects on calf health compared to late group housing.

## Introduction

Calves raised in natural pasture systems are likely to consume forage early in life, but the inclusion of forage in their diets before weaning is controversial because of its potential negative effects on calf performance^1^. Feeding forage to calf starters may provide benefits such as increasing solid feed intake and weight gain^2^, increasing chewing activity^3^, improving the rumen environment and rumen musculature^4,5^, and reducing non-nutritive oral behavior (NNOB)^3,6^. However, because the rumen capacity of young calves is limited, a high proportion of forage may result in lower feed intake, digestibility, and weight gain in young calves^7^. Horvath et al.^8^ describe that providing hay to preweaning group-housed calves resulted in higher total feed intake and improved welfare compared to group-housed calves fed starter alone. Feeding hay with sufficient particle size to dairy calves is thought to be necessary to promote chewing activity and salivary secretion, which increase rumen pH^9,10^ and promote rumen development^11^. The production of saliva, secretion of buffers into the rumen, and the capacity of the rumen wall and papillae to uptake of short-chain fatty acids (SCFA) are involved in the regulation of rumen acidity^12^. In addition, the amount of organic matter and carbohydrates consumed daily and the degradation of carbohydrates are very important factors in maintaining physiological pH levels in the rumen^12^. In dairy calves, accumulation of SCFA due to delayed rumen development can cause persistently low rumen pH (5.5) and, in turn, rumen acidosis^13,14^, resulting in reduced organic matter fermentation^15^. Inclusion of forage in calf starter can help maintain rumen pH^16^ and its effects on feed intake and growth become more apparent as the calf ages^17^. Laarman and Oba^9^ found that hay intake increased rumen pH and that intake of 80 g hay per day reduced the time when rumen pH was below 5.8. When rumen pH was persistently low, hay intake may increase feed intake^17^. This may be more pronounced in group-housed calves, as it has been reported that rearing calves in social housing may result in earlier initiation of feeding, increased intake of solid feed, and sustained intake of solid feed in calves^18^. Several factors can influence the effects of forage feeding on calf intake and growth, including the type of forage (e.g., legumes, grasses, straw, corn silage), the proportion of forage in the calf diet, and the physical form of the calf diet^16,19,20^ as well as the hay quality^21^. For example, feed intake of calves fed alfalfa hay (AH) was higher than different types of forages (ryegrass hay, oat hay, barley straw, triticale silage, corn silage)^16^. Although feeding hay would be needed to prevent rumen acidosis in dairy calves fed ground, pelleted, or poorly textured starters^16,22^, this does not result in the optimal pattern of butyrate and propionate in rumen fermentation^23^. According to the recent review study^1^, calves should be provided with small amounts of high-quality hay such as AH to improve their feed intake and growth rate. In addition, hay feeding is likely to be increased or altered in calves raised in a group housing system.

Keeping calves together during milk feeding promotes normal social behavior and encourages them to mimic their natural behavior during weaning^24,25^, and reduces stress-related behaviors (vocalization) in dairy calves during the weaning transition^25–27^ or after weaning^25^. Several studies have shown that social housing increases competitive success behavior^28^, interactions^29^, and time spent eating, chewing, and rumination after weaning^30^. In addition, calves in group housing have higher weaning weights than calves in individual housing, perhaps as a result of increased DM intake (DMI) during the preweaning period^25,27,31^. Increased DMI is often associated with social learning and social facilitation during feeding^32^.

Animal welfare legislation in Europe (European Council Directive 2008/119/EC, 2008) requires milk-fed veal calves to be given solid feed and raised in groups once they are eight weeks old^33^. Despite animal welfare legislation and all the advantages of rearing calves in group housing, in most dairy farms calves are housed individually during the milk feeding period (reviewed by Costa et al.^18^) and only transferred to group housing after weaning^34^. The main reason for delaying group housing of calves is to reduce disease transmission and the occurrence of behavioral problems, such as cross-sucking, and to provide better feeding and health care for the calf^27,35^. It has been reported that grouped calves had a greater risk of pneumonia and diarrheal disease in the first 3 weeks of life than individual calves^36^. Another study^30^ reported a higher incidence of diarrhea in calves housed in pairs compared to calves housed singly during the third week of life, but not in the following weeks. During the first few weeks of life, the immune system matures, and although all major components of the immune system are present in neonates at birth, many components are not functional until at least 2–4 weeks of age^37^. Therefore, the risk of morbidity and mortality, as well as concern for early grouping, may be reduced if calves are not grouped before 4 weeks of age. Instead of grouping calves after weaning, which is standard practice on most dairy farms, calves can be grouped this way during the suckling period (at 4 weeks of age) without negative health effects and can reap the benefits of early grouping.

Therefore, management practices that contribute to maintaining health, welfare, and improved rumen development and animal performance are beneficial and need to be explored. In addition, the combined effects of group housing and hay feeding on calf growth have not been investigated. Thus, the objective of this study was to investigate the interaction between age at group housing (early = d 28 ± 2, EG vs. late = d 70 ± 2, LG) and age at incremental hay supplementation in starters from 7.5 to 15% of dry matter (DM; early = d 42 ± 2 d, EH vs. late = d 77 ± 2, LH) on growth performance, behavior, health of dairy calves, and development of heifers through first breeding. In this study, we hypothesized that grouping of calves and increasing the amount of hay in the finely ground starter feed from 7.5 to 15% of DM during the pre-weaning period compared to the post-weaning period would improve calf performance, health, and welfare as well as heifer development through first breeding.

## Materials and methods

The study was conducted in a commercial dairy farm (Fazil, Isfahan, Iran) from January 2019 to May 2019. Ethical approval for all procedures involving animals was obtained from the Animal Care and Use Committee of the Isfahan University of Technology before the start of the study. All methods were performed following Iranian Council of Animal Care^38^ regulations. The study complies with ARRIVE guidelines for reporting in vivo experiments and all methods were performed in accordance with the relevant guidelines and regulations.

A total of 64 Holstein dairy calves (41.5 ± 2.10 kg body weight; mean ± SE) were separated from their dams immediately after birth, weighed, and placed in individual pens (1 × 1.5 m) lined with straw. Calves were fed 2.5 l of fresh, pooled, and high-quality colostrum (Brix value > 22) via nipple bottles at each of the first two feedings (i.e., within 30–60 min after birth and 8 h after the first feeding). From the second feeding time until day 3 of life, all calves received colostrum and transition milk twice daily at 0800 and 1600 h. The quality of colostrum was measured using a digital Brix refractometer (PAL-1, Atago Co. Ltd., Bellevue, WA) and discarded if it had a value less than 22 on the Brix scale^39^. If the quality of colostrum was insufficient, frozen-thawed colostrum of sufficient quality was used for the calves. Blood samples were collected by venipuncture from the jugular vein 24 h after the first colostrum feeding, and total serum protein was measured as an indicator of passive transfer of immunity using a Digital Hand-Held Refractometer (VET 360; Reichert Inc., Depew, NY). Only calves with a serum protein level of > 5.5 g/dL were included in this study (all calves in this study had a serum protein level of > 6.6 g/dL).

### Treatments

**Figure 1:**
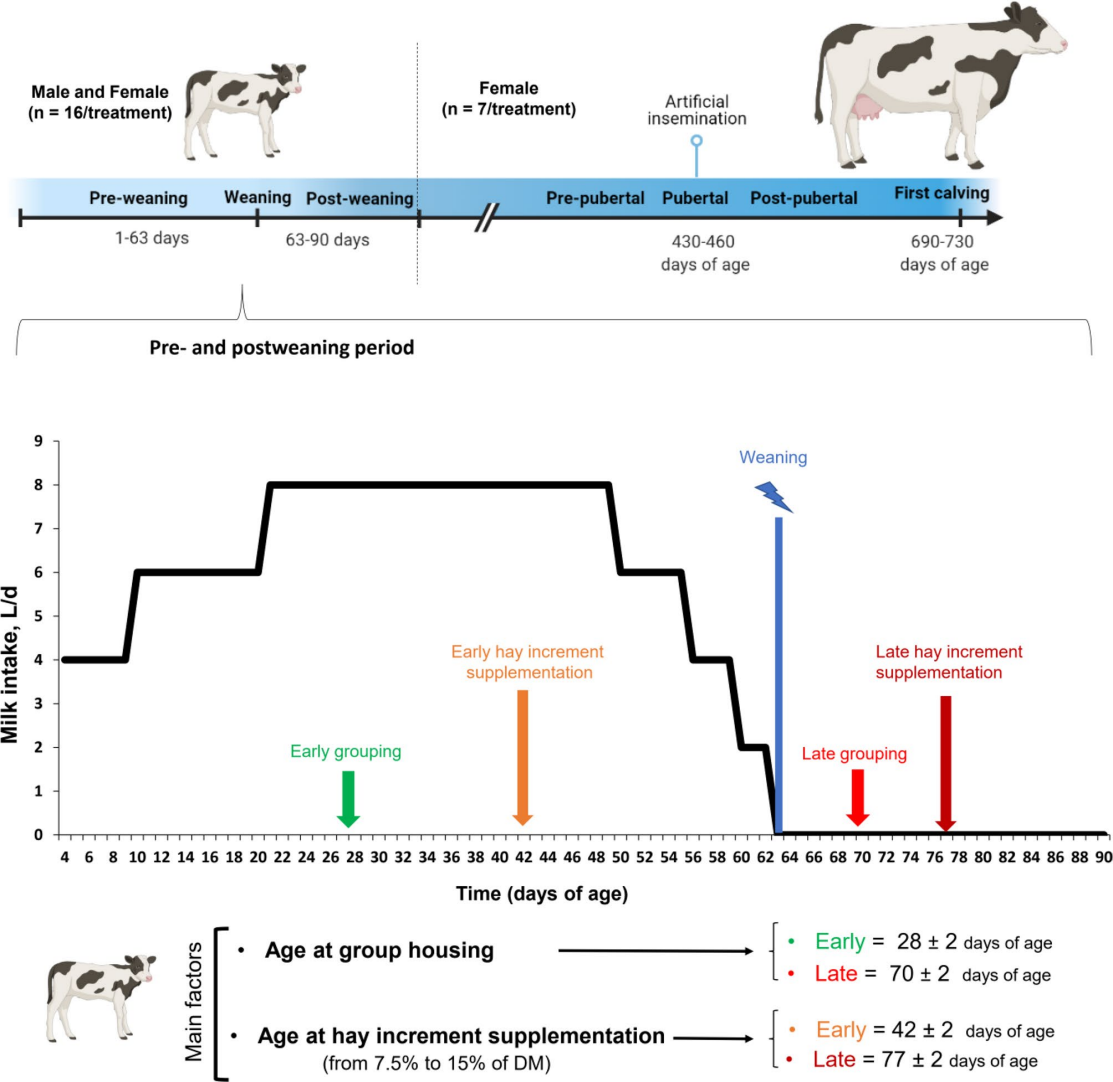
Overview of the animal experiment. A total of 64 calves (n = 16 calves/treatment, 8 male and 8 female) were randomly assigned to 4 treatments in a 2 × 2 factorial arrangement with age at group housing (early = d 28 ± 2, EG vs. late = d 70 ± 2, LG) and age at incremental hay supplementation in calf starters from 7.5 to 15% of DM (early = d 42 ± 2 d, EH vs. late = d 77 ± 2, LH) as main factors (marked with different colors). Treatments included late group housing-late hay increment (LG-LH), late group housing-early hay increment (LG-EH), early group housing-late hay increment (EG-LH), and early group housing-early hay increment (EG-EH). Calves received 4 L/d from day 4 to 9, 6 L/d from day 10 to 20, 8 L/d from day 21 to 49, 6 L/d from day 50 to 55, and 4 L/d from day 56 to 59. Weaning was initiated by limiting milk intake to morning feedings (2 L) from day 60 to 62 of life. Calves were fully weaned at 63 days of age. All calves (female and male) were observed until they were 90 days of age, and heifers were observed until the first calving. Figure created using a web-based program BioRender (https://app.biorender.com).

A total of 64 calves (n = 16 calves/treatment, 8 male and 8 female) were randomly assigned to 4 treatments in a 2 × 2 factorial arrangement with age at group housing (early = d 28 ± 2, EG vs. late = d 70 ± 2, LG; 4 calves per group) and age at incremental hay supplementation in calf starters from 7.5 to 15% of DM (early = d 42 ± 2 d, EH vs. late = d 77 ± 2, LH) as main factors (Fig. 1; created with www.biorender.com). Treatments included late group housing-late hay increment (LG-LH), late group housing-early hay increment (LG-EH), early group housing-late hay increment (EG-LH), and early group housing-early hay increment (EG-EH).

### Feeding and housing management

During the pre-weaning period, all calves were fed pasteurized waste milk twice daily in 2 equal meals (at 0800 and 1600 h). The waste milk used to feed the calves was pasteurized at 63 °C for 35 min. Calves received 4 L/d from day 4 to 9, 6 L/d from day 10 to 20, 8 L/d from day 21 to 49, 6 L/d from day 50 to 55, and 4 L/d from day 56 to 59. Weaning was initiated by limiting milk intake to morning feedings (2 L) from day 60 to 62 of life (Fig. 1). All calves (female and male) were weaned at 63 days of age and observed until 90 days of age. Heifer calves were managed uniformly from 90 days of age until the first calving to evaluate the long-term effects of treatment (Fig. 1). All calves had free access to the diets and clean drinking water throughout the experimental period. The amount of feed offered was adjusted daily to obtain approximately 5 to 10% orts (i.e., the portion of the starter not consumed over a 24-h period). The feed refusal was collected and weighed daily at 0830 h. Alfalfa hay was chopped before the start of the experiment (Golchin Trasher Hay Co., Isfahan, Iran), and two TMRs (Table 1) were prepared with 7.5% and 15% hay of DM, respectively, and stored at room temperature until feeding.

**Table 1:** Ingredients, chemical compositions, and particle size distribution of the experimental diets.

Item	Starter diets	
	7.5% hay	15% hay
**Ingredient (% of DM)**		
Alfalfa hay	7.5	15.0
Corn grain	37.8	34.5
Barley grain	18.4	16.8
Soybean meal (45% CP)	30.4	27.8
Calcium carbonate	1.1	1.1
Wheat bran	1.5	1.5
Sodium bicarbonate	1.0	1.0
Magnesium oxid	0.3	0.3
Vitamin premix^a^	1.0	1.0
Mineral premix^a^	1.0	1.0
**Chemical composition (% of DM)**		
DM	93.9 ± 0.05	93.6 ± 0.02
CP	20.4 ± 0.47	18.2 ± 0.23
NDF	18.3 ± 0.21	23.4 ± 0.13
Ether extract	2.32 ± 0.14	2.03 ± 0.42
Ash	8.13 ± 0.06	8.55 ± 0.15
Starch	35.7 ± 0.35	30.0 ± 0.14
ME^b^ (Mcal/kg)	3.0	2.88
**Particle size distribution**		
4.75 mm	5.35 ± 0.29	12.2 ± 0.33
2.36 mm	10.7 ± 1.08	9.33 ± 0.33
1.18 mm	21.6 ± 0.49	20.4 ± 0.59
0.6 mm	27.7 ± 1.17	29.5 ± 2.04
0.3 mm	26.6 ± 1.47	22.2 ± 3.18
0.15 mm	7.20 ± 1.69	5.72 ± 2.01
Pan	0.60 ± 0.28	0.52 ± 0.26
> 2.36	16.0 ± 1.85	21.5 ± 3.48
< 1.18	83.7 ± 1.88	78.4 ± 3.65
GMPL,^c^ mm	0.93 ± 0.05	1.12 ± 0.06

^a^Contained per kilogram of supplement, 250,000 IU vitamin A, 50,000 IU vitamin D, 1500 IU vitamin E, 2.25 g Mn, 120 g Ca, 7.7 g Zn, 20 g P, 20.5 g Mg, 186 g Na, 1.25 g Fe, 3 g S, 14 mg Co, 1.25 g Cu, 56 mg I, and 10 mg Se.

^b^Calculated according to NRC^40^.

^c^Geometric mean particle length.

All calves were housed in outdoor pens and the space capacity per calf was 3 m2 for all treatment pens. The interior of each pen was bedded with sawdust, which was replaced every day and replenished as needed throughout the study period. All individual pens (1.5 m × 2.0 m) were solid on 3 sides (1.4 m high) with a metal gate at the front corresponding to 2 holes on each gate that allowed calves to access buckets (one for water, one for starter). At each milk feeding, the water buckets were temporarily removed and the milk buckets were placed in the same location in the pen. The fixed individual pens were arranged in rows so that the two rows faced each other. This allowed the individual calves to hear and see the other calves through the openings in the gate, but not to communicate tactilely with other calves. In the group-housed calves, 4 calves were randomly allocated to each pen. Calves were moved to group housing on day of 28 ± 2 d or 70 ± 2 d. In the group pens (3 m × 4 m), animals received water from a water trough (0.15 × 0.8 × 0.25 m width, length, and height, respectively) and solid feed from a feed bunk (0.35 × 1.5 × 0.15 m width, length, and height, respectively). Calves were prevented from stealing each other's milk by placing milk buckets in holes at the front of the group pens.

### Measurements, sampling, and analyses

All calves received the milk offered without refusing it. Feed intake was determined daily by weighing the amounts of feed offered and refused and recorded weekly. Feed intake was recorded for all calves at pen level. Calves were weighed at birth and on days 28, 42, 63, and 90 of the experiment using an electronic scale (model EES-500; Ettehad Inc., Isfahan, Iran) that was calibrated by the manufacturer's representative before the start of the study and once every four weeks. Average daily gain (g/d) was calculated as the difference between BW measured in different periods divided by the number of days between periods at the individual level. Feed efficiency (FE) was calculated as g ADG/g total DMI (liquid feed DMI + starter feed and hay DMI) at the pen level. We collected data on age at first artificial insemination (AI), age at first calving, height at withers at first calving, and BW at first calving. Heifer calves were weighed using an electronic scale two days after the first calving on consecutive days, and height at withers was measured for each heifer.

Milk samples were collected twice weekly and immediately analyzed separately at the farm's Central Milk Testing Laboratory using Milkoscan (Foss Electric, Hillerød, Denmark). The composition of the milk contained 3.23 ± 0.19% fat, 2.91 ± 0.05% CP, 4.35 ± 0.06% lactose, 8.60 ± 0.15% solids not fat (SNF), 11.8 ± 0.17% DM.

Throughout the experiment, samples of diets and orts were collected daily and pooled weekly for analysis. Samples were dried in a forced-air oven at 65 °C for 48 h for the determination of DM and then ground to pass through a 1-mm sieve using a Willey mill (Arthur Thomas Co. Philadelphia, PA). The triply ground samples were analyzed for CP, ether extract, and ash using the methods of AOAC International^41^ and for NDF using heat-stable α-amylase^42^. Starch was hydrolyzed to glucose using a modified glucoamylase method as described by Zhu et al.^43^.

At least 8 representative samples from each TMR were collected and used for particle size distribution. The particle size distribution of the two TMR were measured using a dry sieving technique and an automatic sieve shaker (Sieve Shaker, M. 120, Techno Khak, Khavaran, Tehran, Iran) with sieve diameters of 4.75, 2.36, 1.18, 0.85, 0.60, 0.30, 0.15-mm and a bottom pan (Table 1). Exactly 100 g of each sample was placed on the top sieve in duplicate and the sieve stack was shaken until there was no change in the distribution of the materials (approximately 10 min). The geometric mean particle size and geometric standard deviation were calculated according to the equations of ASABE (Method S319.3)^44^. The ingredients of the TMR, chemical composition, and particle size distribution of the experimental diets are shown in Table 1.

Behavioral data were recorded by direct observation of all calves individually over a 23-h period (between 0800 and 0700 h) on 2 consecutive days after weaning (day 88 to 89 of age). Under the supervision of the authors, three well-trained individuals were assigned to observe the behavior of calves during an experiment, in which they were unaware of the treatments. To facilitate the recording of behaviors, the body parts of the calves were stained. Observers recorded the occurrence of the following behaviors: lying, standing, eating, drinking, rumination, and NNOB. All activities were recorded every 5 min, and it was assumed that each activity continued during the 5-min interval between observations. A period was defined as at least one observation of eating activity after at least 5 min without eating. Meal frequency was defined as the number of bouts during a 23-h period. Meal length (min/meal) was calculated as the time from the start of the first feeding event to an interval between events and averaged for each calf. Inter-feeding event intervals (min) were calculated from the end of one feeding event to the beginning of the next and averaged for each calf. The same method was used to calculate the other behaviors.

Calf health was checked daily throughout the experiment before morning feeding by the farm veterinarian and a member of the research team, as described by Heinrichs et al.^45^. The health check included fecal scoring (1 = normal; 2 = soft to loose; 3 = loose to watery; 4 = watery, mucous, and slightly bloody; and 5 = watery, mucous, and bloody), general appearance (1 = normal and alert; 2 = ears drooping; 3 = head and ears drooping, dull eyes, slightly lethargic; 4 = head and ears drooping, dull eyes, lethargic; and 5 = severely lethargic) and respiratory assessment (1 = normal, 2 = mild cough, 3 = moderate cough, 4 = moderate to severe cough, 5 = severe and chronic cough). Fecal scores were recorded by observing the tail-head region and the rear end of the calf soiled with feces or by stimulating the rectum of each calf. Fecal score was categorized as the number of days with a fecal score ≥ 3. General appearance and respiratory scores were categorized as the number of days with a score ≥ 2. These categories were reported as days with an abnormal fecal score, respiratory score, and general appearance and were scored by the veterinarian to verify the diagnoses of diarrhea and pneumonia. The diagnoses of diarrhea and pneumonia and the treatment of the calves confirmed by the veterinarian followed standard operating procedures at Fazil Agri.

Calves with diarrhea (fecal score ≥ 3) were first treated with an oral electrolyte solution (4 L/d per calf in 2 equal meals for 3 consecutive days; Damyaran Arak Vet. Pharmaceutical Co., Saveh, Iran). When a calf's body temperature was ≥ 39.4 °C, 2 mL of flunixin meglumine (flunixin 5%; 5 mL per calf for the first 3 consecutive days; Razak Laboratories Co.) and 5 mL of amoxicillin (Norbrook Laboratories Ltd., Newry, Northern Ireland) were administered intramuscularly for 3 consecutive days. To treat pneumonia, calves were given florfenicol (F-nex 300; 6 mL per calf for 5 consecutive days; Razak Laboratories Co., Karaj, Iran) and flunixin meglumine (Flunixin 5%; 5 mL per calf for 5 consecutive days; Razak Laboratories Co.) When the calf's body temperature was ≥ 39.5 °C, oxytetracycline was administered in addition to the high protocol (Tenaline 20% L. A.; 8 mL per calf on day 1 and 3; RooyanDarou Pharmaceutical C0., Semnan, Iran).

### Data analysis and statistics

Before analysis, all data were checked for normality by evaluating the Shapiro–Wilk statistic using the UNIVARIATE procedure of SAS. Data that were not normally distributed were log-transformed. The effects of treatments on starter intake, total DMI, nutrient intake (NDF, ME, starch, and CP), and FE were tested by obtaining the mean value of the pen (based on the mean of the 4 calves per pen in the treatment) in each week of the experiment. Data for starter intake, total DMI, nutrient intake (weeks 1 to 13), and FE (weeks 1 to 13) were analyzed using the MIXED procedure from SAS (SAS 9.4, SAS Institute Inc., Cary, NC) with time (week) as repeated measures at ANOVA. The model included fixed effects age at group housing, age at increasing hay level, main effect interaction, time, and age in group housing × age at increasing hay level × time. The random effect was a pen. Covariance structures of compound symmetry and autoregressive (type 1) were tested for within-subject measurements, and the best-fit structure was defined using Bayesian information criteria.

Data for behavior [time (min/23 h), bout length (min/bout), bout interval (min)] were obtained from each calf and analyzed using the MIXED procedure of SAS. The model consisted of age at group housing, age at incremental hay supplementation in starters, interaction of main effects and sex as fixed effects, and pen nested within age at group housing × age at incremental hay supplementation as random effects.

Data for BW and ADG were also obtained from each calf and subjected to repeated measures over time using the MIXED procedure of SAS (SAS 9.4, SAS Institute Inc., Cary, NC) ANOVA. The model included fixed effects of sex, age at group housing, age at incremental hay supplementation, time, and their interactions. The random effect was calf nested age at group housing × age at incremental hay supplementation × pen. Breeding data were analyzed with ANOVA using the MIXED procedure of SAS with age at group housing and age at increasing hay level as fixed effects. Significance was indicated at *P* ≤ 0.05, and trends were indicated when 0.05 ≤ *P* ≤ 0.10.

Models for incidence of diarrhea, pneumonia, need for medication, and general appearance (≥ 2) were analyzed by logistic regression using a binomial distribution in the GLIMMIX procedure in SAS. Odds ratios were used to compare the probability for calves in each main experimental effect (age at group housing and age at incremental hay supplementation) to experience any event. Frequency and duration of diarrhea, pneumonia, administration of medication, and number of days with general appearance (≥ 2) were analyzed with a Poisson distribution using the GLIMMIX procedure in SAS (version 9.4).

## Results

### Dietary properties, and particle size distribution

Table 1 shows the ingredient composition, chemical composition, and particle size distribution of the experimental diets. As indicated, the concentrations of crude protein (CP; from 20.4 to 18.2%), metabolizable energy (ME; from 3 to 2.88 Mcal/kg), and starch (from 35.7 to 30.0%) were slightly lower in the TMR with 15% AH due to the higher content of chopped AH in the calf starters. However, the addition of AH increased the NDF content of the starter diet from 18.3 to 23.4%. The percentage of particles retained on the 2.36-mm sieves was greater in a diet containing 15% AH than in a diet containing 7.5% AH (21.5 vs. 16.0%), resulting in a greater geometric mean particle length (GMPL) of 1.12 mm versus 0.93 mm. However, the 7.5% AH feed contained more particles retained on the 1.18-mm sieves (i.e., a sum of 1.18, 0.6, 0.3, 0.15 mm, and pan) than the 15% AH feed with a percentage of 83.7 versus 78.4.

### Nutrient intake, growth, and development of heifers through first breeding

**Figure 2:**
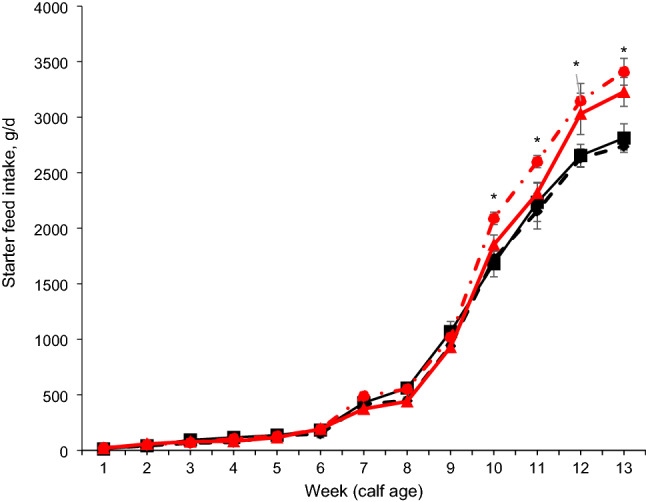
Starter feed intake of calves (n = 16 calves/treatment, 8 male and 8 female) with one of the following treatments: Late grouping-late hay increment (LG-LH; black filled square), late grouping-early hay increment (LG-EH; black filled diamond), early grouping-late hay increment (EG-LH; red filled triangle), and early grouping-early hay increment (EG-EH; red filled circle) during the total period of study (from 1 to 13 wk of age). Data are presented as means ± SEM. Asterisk indicate a significant difference (*P < 0.05) between groups at a given time point.

**Figure 3:**
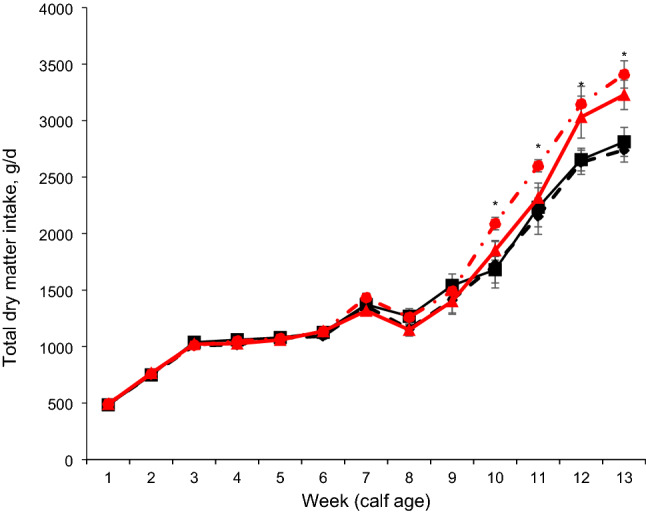
Total dry matter intake of calves (n = 16 calves/treatment, 8 male and 8 female) with one of the following treatments: Late grouping-late hay increment (LG-LH; black filled square), late grouping-early hay increment (LG-EH; black filled diamond), early grouping-late hay increment (EG-LH; red filled triangle), and early grouping-early hay increment (EG-EH; red filled circle) during the total period of study (from 1 to 13 wk of age). Data are presented as means ± SEM. Asterisk indicate a significant difference (*P < 0.05) between groups at a given time point.

**Figure 4:**
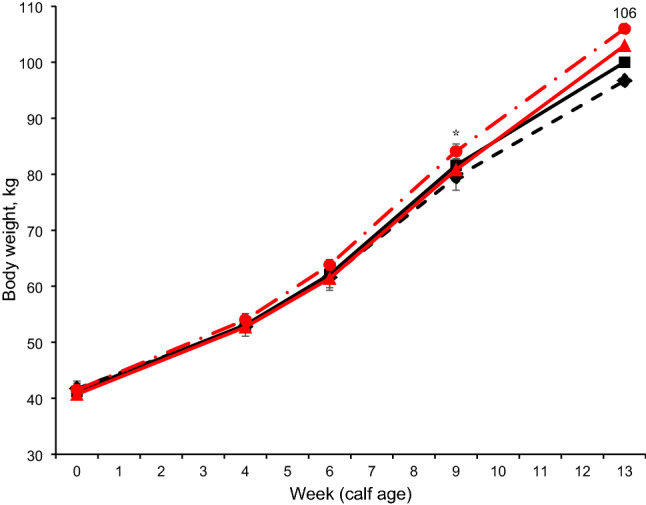
Body weight of calves (n = 16 calves/treatment, 8 male and 8 female) with one of the following treatments: Late grouping-late incremental hay (LG-LH; black filled square), late grouping-early incremental hay (LG-EH; black filled diamond), early grouping-late incremental hay (EG-LH; red filled triangle), and early grouping-early incremental hay (EG-EH; red filled circle) during the total period of study (from 1 to 13 wk of age). Data are presented as means ± SEM.

No interaction was found between age at group housing and age at incremental hay supplementation in starters (Table 2) on starter intake, total DMI, NDF intake, starch intake, ADG and feed efficiency (FE). Nutrient intake and growth performance were not affected by age at the incremental hay supplementation level. But the EH calves had higher NDF intake at overall period and weeks 7, 8, 9, 10, and 11 (*P* < 0.05) than the LH calves. The EG calves had higher (*P* = 0.01) starter intake (1022 vs. 905 g/d), TDMI (1566 vs. 1450 g/d), ME intake (5.92 vs. 5.58 Mcal/kg), NDF intake (229 vs. 202 g/d), CP intake (191 vs. 170 g/d), starch intake (316 vs. 282 g/d), and ADG (770 vs. 716 g/d; *P* = 0.02) compared to the LG calves throughout the experimental period (Table 2). The EG calves had a higher intake of starter feed (Fig. 2), total DM (Fig. 3), and ME (Supplemental Fig. S1A) compared to LG calves at weeks 10, 11, 12, and 13 of the experiment. In addition, EG calves had greater starch intake and CP intake at weeks 10 and 11 (trend; *P* < 0.10) and weeks 12, 13 (*P* < 0.01) compared to LG calves (Supplemental Fig. S1B, C). NDF intake was greater in EG calves than in LG calves at weeks 11, 12, and 13 (Supplemental Fig. S1D; *P* < 0.05). Calf BW at 90 days (104 vs. 98.4 kg, *P* < 0.01) and total BW (76.0 vs. 72.9 kg, *P* = 0.02) were greater in EG calves than in LG calves (Fig. 4).

**Table 2:** Effects of age at group housing [G; late grouping (LG) vs. early grouping (EG)] and age at incremental hay supplementation [late hay increment (LH) vs. early hay increment (EH)] on starter dry matter intake (DMI), nutrient intakes, feed efficiency, ADG (weeks 1–13, n = 16 per treatment), and development of heifers through first breeding (n = 7 calves per treatment).

Item	Treatments				SEM	*P* value					
	LG-LH	LG-EH	EG-LH	EG-EH		Group housing (G)	Hay increment (H)	G × H	Period (P)	G*P	H*P
Starter DMI, g/d	924	887	978	1066	40.1	0.01	0.53	0.14	< 0.01	< 0.01	0.34
Neutral detergent fiber intake, g/d	199	206	212	247	8.89	0.01	0.03	0.13	< 0.01	< 0.01	< 0.01
Starch intake, g/d	296	268	311	322	12.6	0.01	0.50	0.14	< 0.01	< 0.01	0.15
Crude protein intake, g/d	177	164	186	196	7.52	0.01	0.83	0.14	< 0.01	< 0.01	0.78
Metabolizable energy intake, Mcal/kg	5.66	5.51	5.81	6.03	0.11	0.01	0.74	0.14	< 0.01	< 0.01	0.97
Total DMI (milk + starter), g/d	1469	1431	1522	1611	40.1	0.01	0.53	0.14	< 0.01	< 0.01	0.92
Average daily gain, g/d	740	686	752	785	26.8	0.02	0.83	0.16	< 0.01	0.06	0.68
Feed efficiency, ADG/DMI	0.56	0.54	0.56	0.57	0.01	0.47	0.78	0.49	< 0.01	0.48	0.49
**Development of heifers through first breeding**											
Age at first artificial insemination, d	443	460	442	437	16.5	0.47	0.69	0.52	–	–	–
Average age at first calving, d	717	729	693	711	19.3	0.28	0.46	0.85	–	–	–
Average wither height at first calving, cm	145	143	146	144	1.94	0.63	0.34	0.85	–	–	–
Average weight at first calving, kg	584	554	567	558	16.8	0.70	0.24	0.54	–	–	–

No differences were observed among the treatments for age at first AI, age at first calving, withers height at first calving, and weight at first calving (Table 2).

### Health criteria

Table 3 shows the logistic models for the occurrence of general appearance (score ≥ 2), diarrhea (score ≥ 3), pneumonia, and needs for medication during the period (week 1 to 13). The EG or EH did not affect the incidence of general appearance (score ≥ 2), diarrhea, and pneumonia. In addition, the likelihood of administering medication for diarrhea and pneumonia was not affected in EG calves compared to LG calves or EH calves compared to LH calves.

**Table 3:** Logistic model for general appearance ≥ 2,^a^ diarrhea ≥ 3,^b^ pneumonia, and medication occurrence during the overall (week 1–13) period as influenced by late grouping (LG) or early grouping (EG) and late hay increment (LH) or early hay increment (EH) to Holstein calves.

Variable and comparison	Coefficient	SEM	Odds ratio^c^	95% CI	*P* value
**General appearance**					
LG versus EG	0.01640	0.12	1.01	0.80–1.27	0.88
LH versus EH	− 0.09910	0.11	0.90	0.72–1.13	0.39
**Diarrhea occurrence**					
LG versus EG	− 0.00908	0.11	0.99	0.79–1.23	0.93
LH versus EH	0.1803	0.12	1.19	0.96–1.49	0.10
**Pneumonia occurrence**					
LG versus EG	− 0.03917	0.16	0.96	0.70–1.32	0.80
LH versus EH	− 0.03651	0.17	0.96	0.70–1.32	0.82
**Medication occurrence**					
Diarrhea					
LG versus EG	− 0.01963	0.12	0.98	0.76–1.26	0.87
LH versus EH	− 0.1010	0.12	0.90	0.70–1.16	0.43
**Pneumonia**					
LG versus EG	− 0.1330	0.18	0.87	0.61–1.24	0.46
LH versus EH	− 0.1952	0.19	0.82	0.57–1.17	0.28

^a^1 = normal and alert; 2 = ears drooped; 3 = head and ears drooped, dull eyes, slightly lethargic; 4 = head and ears drooped, dull eyes, lethargic; 5 = severely lethargic^45^.

^b^1 = normal; 2 = soft to loose; 3 = loose to watery; 4 = watery, mucous, and slightly bloody; 5 = watery, mucous, and bloody^45^.

^c^The odds ratio (OR) indicates the probability of having diarrhea or pneumonia, or needing medication for the experimental treatments (LG vs. EG; and LH vs. EH).

If the OR is > 1, the treatment in the comparison is more likely to have diarrhea or pneumonia or to be medicated than the other treatment by a factor of the difference above 1. If the OR is < 1, the treatment has a lower probability of occurrence than the other treatment.

Table 4 shows Poisson regression for the frequency and number of days with general appearance (score ≥ 2), diarrhea (score ≥ 3), and pneumonia, and the number of days treated with medication for both diarrhea and pneumonia. We observed no difference among the treatments for these scores during the experiment.

**Table 4:** Poisson regression for days with general appearance ≥ 2,^a^ frequency and duration of diarrhea ≥ 3, ^b^ pneumonia, and days medicated during the overall (week 1–13) period as influenced by treatments containing age at group housing [G; late grouping (LG) vs. early grouping (EG)] and age at incremental hay supplementation [late hay increment (LH) vs. early hay increment (EH)].

Item	Treatments				SEM	*P* value		
	LG-LH	LG-EH	EG-LH	EG-EH		Group housing (G)	Hay increment (H)	G × H
Days with general appearance ≥ 2	4.61	5.75	5.20	6.01	0.10	0.45	0.10	0.73
**Diarrhea**								
Frequency, times diagnosed	0.85	0.83	0.95	1.12	0.24	0.45	0.78	0.71
Duration, d	3.87	4.41	4.17	4.31	0.12	0.83	0.51	0.70
Medicated, d	3.90	4.25	4.23	4.39	0.12	0.64	0.62	0.84
**Pneumonia**								
Frequency, times diagnosed	0.98	1.10	0.75	1.32	0.22	0.87	0.18	0.37
Duration, d	2.37	2.27	1.86	2.53	0.16	0.69	0.45	0.31
Medicated, d	2.09	2.13	1.57	2.58	0.17	0.79	0.15	0.19

^a^1 = normal and alert; 2 = ears drooped; 3 = head and ears drooped, dull eyes, slightly lethargic; 4 = head and ears drooped, dull eyes, lethargic; 5 = severely lethargic^45^.

^b^1 = normal; 2 = soft to loose; 3 = loose to watery; 4 = watery, mucous, and slightly bloody; 5 = watery, mucous, and bloody.

### Behavior

Description of the recorded behaviors is presented in Table 5. The behavioral activities are shown in Table 6. After weaning, we found no effect of age at incremental hay supplementation level and no interaction between main effects on the behavioral patterns (time, bout frequency, bout length, and bout interval) of eating, drinking, rumination, lying, standing, and NNOB. Also, the activities of drinking, rumination, and lying down were not affected by age in the group housing treatment. However, time spent eating (234 vs. 212 min; *P* = 0.03), frequency of eating (16.4 vs. 15.0 no./23 h; *P* = 0.02) increased and interval of bout eating decreased in EG calves compared to LG calves (74.3 vs. 85.3 min; *P* = 0.02), but not eating bout length. Early grouping of calves did not affect lying pattern, but the number of standing bouts per 23 h (16.7 vs. 19.4 no./23 h; *P* = 0.05) was lower in EG calves than in LG calves. Time spent in NNOB behavior also tended to be lower in EG calves (25.5 vs. 32.9 min; *P* = 0.09) than in LG calves.

**Table 5:** Description of the recorded behaviors by Leruste et al.^46^ and Abdelfattah et al.^47^.

Behaviors	Description
Standing	Standing with all four feet on the ground without any purposeful activity
Lying	Lying on flank or sternum with head held in a raised position or down without rumination
Eating	Ingesting starter from feed bunk or head was in the feed bunk and ingesting milk
Drinking	Drinking water from water trough
Ruminating	Irregular, repetitive chewing without discernible food in the mouth either lying or standing
Non-nutritional oral behavior	Biting, nibbling, sucking or licking any pen structures, except for feed; manipulating a penmate including sucking or bitting a part of the body of a penmate; consuming bedding materials, and calf rolling its tongue outside the mouth

**Table 6:** Effects of age at group housing [late grouping (LG) vs. early grouping (EG)] and age at incremental hay supplementation [late hay increment (LH) vs. early hay increment (EH)] on behaviors activities after weaning (d 88 and 89) in dairy calves (n = 16 calves per treatment).

Item	Treatments				SEM	*P* value		
	LG-LH	LG-EH	EG-LH	EG-EH		Group housing (G)	Hay increment (H)	G × H
**Eating**								
Time, min/23 h	213	211	236	233	9.39	0.03	0.82	0.94
Meal frequency, no./23 h	14.8	15.3	16.3	16.5	0.54	0.02	0.55	0.68
Meal length, min/bout	14.4	13.7	14.6	14.2	0.58	0.54	0.41	0.83
Meal interval, min	86.1	84.6	72.1	76.6	4.49	0.02	0.69	0.60
**Drinking**								
Time, min/23 h	13.1	17.7	16.4	15.0	2.26	0.82	0.62	0.17
Frequency, no./23 h	2.75	3.17	3.28	2.88	0.42	0.70	0.99	0.10
Bout length, min/bout	4.76	5.35	4.97	5.22	0.52	0.82	0.45	0.21
Bout interval, min	408	286	235	358	73.3	0.50	0.62	0.26
**Ruminating**								
Time, min/23 h	372	362	358	403	22.5	0.56	0.45	0.24
Frequency, no./23 h	18.7	18.0	17.4	18.3	0.60	0.42	0.90	0.23
Bout length, min/bout	19.9	20.1	20.6	22.1	0.81	0.11	0.33	0.44
Bout interval, min	55.5	60.2	56.4	53.4	3.25	0.33	0.84	0.21
**Lying**								
Time, min/23 h	566	560	563	543	18.6	0.60	0.49	0.69
Frequency, no./23 h	21.3	22.0	22.4	21.3	0.96	0.84	0.82	0.34
Bout length, min/bout	26.6	25.5	25.2	25.5	0.96	0.48	0.67	0.44
Bout interval, min	35.4	30.6	32.5	33.5	2.06	0.80	0.36	0.15
**Standing**								
Time, min/23 h	190	200	187	164	11.6	0.11	0.60	0.18
Frequency, no./23 h	19.4	19.4	17.8	15.7	1.19	**0.05**	0.41	0.38
Bout length, min/bout	9.79	10.2	10.5	10.4	0.36	0.28	0.65	0.37
Bout interval, min	68.8	67.1	72.9	81.1	5.63	0.12	0.60	0.50
**NNOB**								
Time, min/23 h	31.6	34.3	24.6	26.5	4.08	0.09	0.58	0.91
Frequency, no./23 h	5.12	5.14	4.50	4.39	0.48	0.24	0.90	0.87
Bout length, min/bout	6.17	6.68	5.48	6.04	0.64	0.22	0.27	0.95
Bout interval, min	201	142	165	180	49.1	0.99	0.28	0.88

## Discussion

The present study examined the effects of age at group housing and age at incremental hay supplementation levels and their interaction on growth performance, behavior, health of dairy calves, and development of heifers through first breeding.

### Age at incremental hay supplementation levels

Dairy calves on some commercial farms have no access to forage during the weaning period, a practice that is contrary to natural grazing. Grazing nursing calves begin to become increasingly dependent on forage from 60 to 90 days of age, depending on the amount of milk they consumed^48^. According to Wu et al.^49^, there were no differences in feed intake, weight gain, or rumen development in calves fed alfalfa or oat hay on day 3 or day 15 of age. Other studies found differential effects depending on the age at which AH^50^ and oat hay^51^ were fed to calves. As noted in both studies, DMI and growth performance improved when forage was provided, with the greatest improvements in growth performance and rumen development occurring in calves offered hay beginning at 2 weeks of age rather than at 4 or 6 weeks of age. As a result of these studies^50,51^, AH or oat hay should be included in the diet of dairy calves during the second week of life or after birth to improve DMI and ADG. In the current study, we hypothesized that increasing the amount of hay in the finely ground starter feed from 7.5 to 15% of DM during the pre-weaning period compared to the post-weaning period would improve calf performance and welfare as well as heifer development through first breeding. In the experiment, we selected hay levels of 7.5% and 15% of DM in the calf starter diets, which corresponded to the range of hay levels commonly observed or suggested in previous studies (e.g., < 10%; Castells et al.^4,52^, and > 10%; Imani et al.^16^). Our results showed that the age at which incremental hay supplementation was administered did not affect starter feed intake, growth (BW, ADG, and FE), or development of heifer through the first breeding. Our findings are in line with the findings of Coverdale et al.^53^, who found that giving 7.5% or 15% hay to individual calves during the preweaning period did not affect feed intake, BW, ADG, body measurements, and FE. However, in their study, all calves of the same age were fed different amounts of hay from the first week of life. Since intake of a high amount of milk during the preweaning period resulted in low consumption of solid feed^54^, no effect of different hay level was probably observed when starter intake was restricted. In our study and the study by Coverdale et al.^53^, the average feed intake (starter and hay) during the preweaning period was low at about 270 and 295 g/d, respectively. The results indicate that feeding hay at a rate of 7.5% of DM during the pre-weaning period was sufficient to drive rumen development improvements, as indicated by the fact that there was no difference in growth performance.

In the current study, calf behaviors (eating, ruminating, drinking, lying, and standing) along with overall health status (general appearance, diarrhea, pneumonia, and medication occurrence) did not differ with age at incremental hay supplementation level. It is likely that providing enough hay (7.5% of DM) in the starter diets from an early age explains the lack of effect of age at incremental hay supplementation levels on calf health and behavior.

### Age at group housing

In the current study, starter feed intake and total DMI were greater in EG calves than LG calves throughout the experimental period, especially after weaning. Costa et al.^24^ reported that early pair housing (6 days) increased feed intake and BW of calves throughout the experimental period than late pair housing (43 days). Age of pairing (5 vs. 28 d) or group housing (birth vs. 3 weeks) did not affect solid feed intake in the Bolt et al.^26^ and Tapki^55^ studies. The increased solid feed intake in EG calves compared to LG calves after weaning could be due to the beneficial effects of social housing, such as better coping with weaning stress^25^, behavioral and feeding flexibility^56^, and increased feed intake in a competitive feeding environment^28,29^. In addition, the greater feed intake in this study is consistent with findings that EG calves spend more time eating after weaning than LG calves.

In the current study, EG calves had greater final BW and ADG than LG calves throughout the period. Abdelfattah, et al.^57^ and Bolt et al.^26^ reported that age at grouping (3, 7, and 14 days old) or pairing (5 vs. 28 d) did not affect final BW, or ADG. However, Costa et al.^24^ reported that average daily gain was not different between early- or late-paired calves during the preweaning period, but early-paired calves had higher ADG than late paired calves during the weaning period. Previous studies observed lower feed intake and weight gain in group-housed calves^58^, higher concentrate intake, and no significant increase in weight gain in group-housed calves^25^, or higher feed intake and weight gain^24,31,59^. In this study, ADG was greater in EG calves than in LG likely due to greater feed intake, and ME intake after weaning and throughout the period. Solid feed intake likely became more important for growth after 10 weeks of age in the current study when calves were weaned from milk. Differences between studies may be related to experimental methodology or management practices. In the current study, we found no differences in heifer development (age at first AI, age at first calving, withers height at first calving, or weight at first calving) through the first breeding when EG calves were compared with LG calves. The lack of differences could be due to the small number of heifers per treatment.

Farmers often avoid early grouping of calves because of adverse health effects. However, in the present study, no evidence of health differences was found between EG calves and LG calves. This was similar to Bolt et al.^26^ who found no differences in fecal and respiratory scores for calves grouped at 5 or 28 days of age, and Abdelfattah et al.^57^ who reported no effects of early (d 3) grouping of calves compared to grouping at 7 and 14 days of age on health scores. These results suggest that there might be little association between age in group housing and calf health and that many other management practices influence disease risk, including hygiene, bedding management, colostrum practices and immunity, milk feeding methods, environmental management, health monitoring, and disease diagnosis, ventilation and nutrition (reviewed by Costa et al.^18^), age of contacting calves, space per calf, and group size^60^. In the present study, all calves achieved a satisfactory serum IgG level (10 mg/mL) by consuming 13% of their BW of colostrum within 8 h of birth, which was realized with a higher initial total blood protein (6.6 g/dL). Bedding was also replaced regularly (every day), space was 3 m^2^ per calf, the age difference between calves per pen (± 2 d), and group size was small (4 calves per pen). Therefore, by controlling the above practices, the health problems of dairy calves in early and late groups can be effectively optimized.

The increase in eating time during the post-weaning period in EG calves was consistent with the increase in feed intake during this period. In addition, EG calves exhibited higher meal frequency along with shorter meal intervals compared to LG calves. Previous studies reported that calves in group housing spend more time at the feeder after weaning^25^ or that feeding time decreased in calves in pair housing during the weaning period (7–8 weeks) and after weaning (9 weeks), probably due to the increased feeding rate in a competitive feeding environment^30^. In these studies, calves were housed in singles or pairs during the milk feeding period (from birth), then mixed and moved to groups of 6 calves after weaning.

Our results show that EG and LG calves spent similar lying and standing times. It has been reported that lying behavior can be influenced by housing system or social partners^6,61^ and pen size^61^. A complex of lying behaviors, including total lying time, number of lying bouts, the average duration of bouts, and laterality, can indicate how the animal interacts with its environment and is an important indicator of animal welfare^62^. Calves housed in the group pen early in life reduced standing frequency than LG calves which indicates less restlessness in EG calves^62^.

In the current study, the NNOB decreased with age in all calves, likely due to an increase in time spent feeding and rumination^6^. We found that EG calves tended to spent less time on NNOB than LG calves. This result suggests that LG calves are likely to be less comfortable than EG calves. This is because it has been reported that the expression of NNOB may be a sign of reduced welfare due to the mismatch of environmental and management characteristics with the behavioral and physiological needs of the animal^46^. Also, reduced NNOB in EG calves may be related to more feed intake and time spent eating. This is because Margerison et al.^63^ stated that feed intake could provide a substitute stimulus to reduce NNOB in calves.

## Conclusion

In the current study, no interactions between age at group housing and age at incremental hay supplementation in calf starters on starter feed intake, calf performance, health, and behavior as well as heifer development through first breeding were observed, which was contrary to our hypothesis. Our results showed that the age at which incremental hay supplementation was administered did not affect starter feed intake, growth (BW, ADG, and FE), or development of heifer through the first breeding (age at first AI, age at first calving, withers height at first calving, and weight at first calving). Regarding the age at group housing, nutrient intakes (DM, ME, CP, NDF, and starch), ADG, and final BW improved in EG compared to LG calves. Also, the frequency of standing decreased and time and frequency of eating increased in early group calves compared to late group calves. Overall results indicate an improvement in growth performance with no adverse effects on calf health for early group housing compared to the late grouping of dairy calves.

## Supplementary Information

Supplementary Figure S1.
