# Supplementary material for: Effects of group housing and incremental hay supplementation in calf starters at different ages on growth performance, behavior, and health
Source: Sci Rep. 2022 Feb 24;12:3190. doi: 10.1038/s41598-022-07210-7 (PMC8873488; doi:10.1038/s41598-022-07210-7)
Supplement: Supplementary file 1 — Supplementary Figure S1. [file 41598_2022_7210_MOESM1_ESM.docx]

**Supplementary Information for**

**Effects of group housing and incremental hay supplementation in calf starters at different ages on growth performance, behavior, and health**

**Fatemeh Ahmadi^1^, Ebrahim Ghasemi^1^, Masoud Alikhani^1^, Majid Akbarian-Tefaghi^1^ & Morteza Hosseini Ghaffari^2^***

^1^ Department of Animal Sciences, College of Agriculture, Isfahan University of Technology, Isfahan 84156–83111, I. R. Iran. ^2^ Institute of Animal Science, Physiology & Hygiene Unit, University of Bonn, 53115 Bonn, Germany. Correspondence and requests for materials should be addressed to (email: morteza1@uni-bonn.de)

| A |
| --- |
| 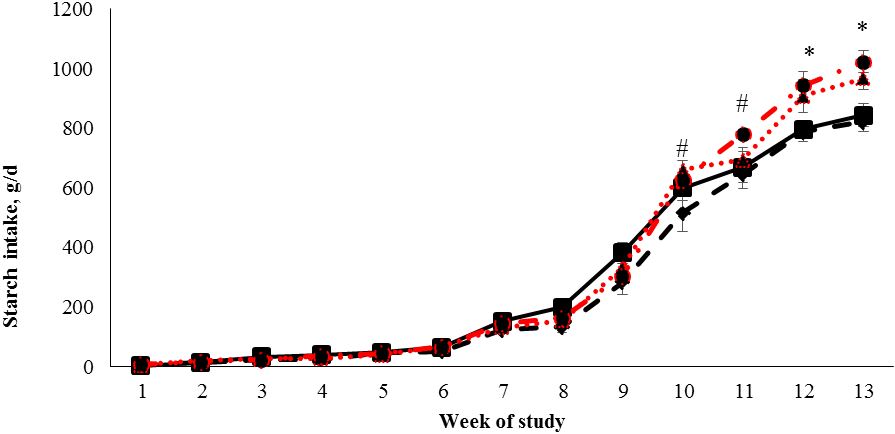 *Overall*  *Grouping P = 0.12*  *Hay treatment P = 0.29*  *Grouping * Hay P = 0.12*  *Time P < 0.01*  *Grouping × Hay × Time P = 0.71* |
| B |
|  |
| C |
|  |
| D |
|  |

**Supplemental Figure S1.** (A) starch intake, (B) crude protein (CP) intake, (C) neutral detergent fiber (NDF) intake, and (D) metabolizable energy **(**ME) intake of calves (n = 16 calves/treatment, 8 male and 8 female) with one of the following treatments: Late grouping-late hay increment (LG-LH; ■), late grouping-early hay increment (LG-EH; ⬥), early grouping-late hay increment (EG-LH; ⯅), and early grouping-early hay increment (EG-EH; ⦁) during the total period of study (from 1 to 13 of age). Data are presented as means ± SEM. For each time point, * denotes significant differences (p <0.05) for housing effects, # denotes significant trend differences (0.05 <p <0.10) for housing effects, $ denotes significant differences (p <0.05) for forage effects.
